# Biomedical Perspective of Electrochemical Nanobiosensor

**DOI:** 10.1007/s40820-015-0077-x

**Published:** 2015-12-21

**Authors:** Priti Singh, Shailendra Kumar Pandey, Jyoti Singh, Sameer Srivastava, Sadhana Sachan, Sunil Kumar Singh

**Affiliations:** 1 Department of Biotechnology, Motilal Nehru National Institute of Technology, Allahabad, Uttar Pradesh 211004 India; 2 Department of Chemical Engineering, Motilal Nehru National Institute of Technology, Allahabad, Uttar Pradesh 211004 India

**Keywords:** Biomarkers, Point-of-care detection, Nanomaterials, Electroactive species, Potentiometric, Amperometric, Implantable

## Abstract

Electrochemical biosensor holds great promise in the biomedical area due to its enhanced specificity, sensitivity, label-free nature and cost effectiveness for rapid point-of-care detection of diseases at bedside. In this review, we are focusing on the working principle of electrochemical biosensor and how it can be employed in detecting biomarkers of fatal diseases like cancer, AIDS, hepatitis and cardiovascular diseases. Recent advances in the development of implantable biosensors and exploration of nanomaterials in fabrication of electrodes with increasing the sensitivity of biosensor for quick and easy detection of biomolecules have been elucidated in detail. Electrochemical-based detection of heavy metal ions which cause harmful effect on human health has been discussed. Key challenges associated with the electrochemical sensor and its future perspectives are also addressed.

## Introduction

**Fig. 1:**
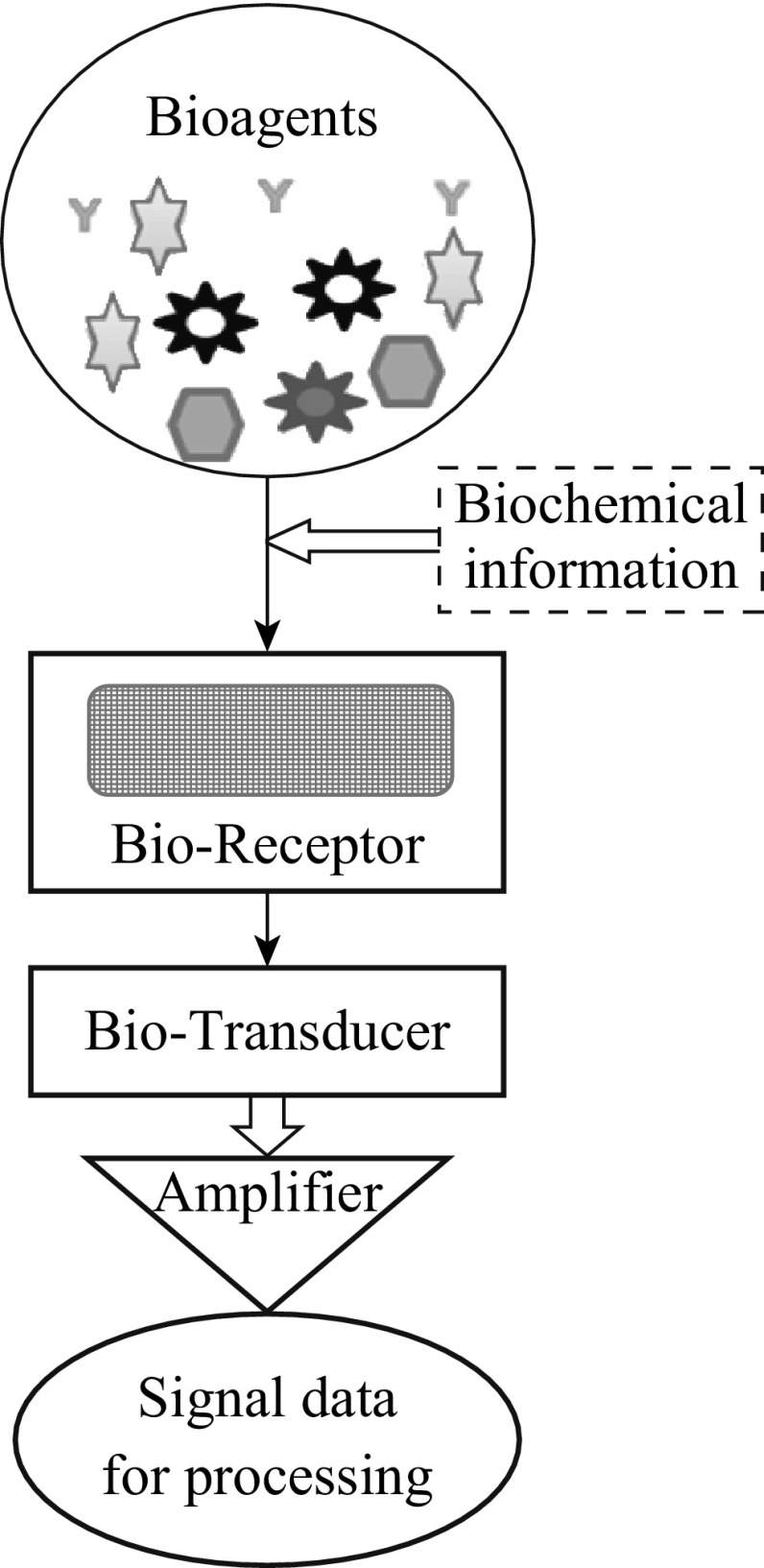
Basic principle of biosensor

**Fig. 2:**
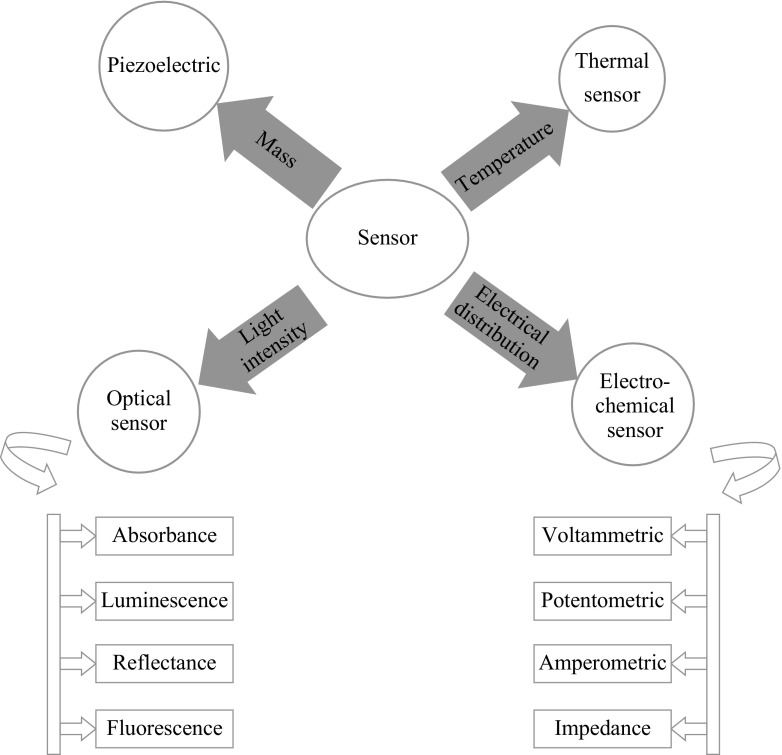
Schematic showing classification of biosensor

Chemical sensor converts information generated from chemical reaction of analytes into an analytical signal by utilizing the physical property of the system investigated [1]. These chemical sensors have vast application in industries for process control and monitoring in safety, environmental protection, detection of biochemical agents, drug development, in-home medical diagnosis and chemical warfare. Analytical signal obtained from the biochemical process are regarded as biosensor. These sensors have great potential for monitoring environmental hazards as well as in health care. Biosensor is a device which integrates a biological recognition element into a transducer. A schematic of the basic principle of biosensor shown in Fig. 1 shows biomolecules incorporated into a solid matrix that holds the sensing bio-analyte. Components of the sensor like electrodes, and intermediate matrix between the recognition layer and transducer play an important role in defining the stability, selectivity and specificity of biosensor [2]. Based on the principle of transducer, biosensors may be classified as shown in Fig. 2. The two major transduction mechanisms like optical and electrochemical sensors are, respectively, based on the light intensity and electrical distribution that play a vital role in a majority of the available biosensors. Among these, the electrochemical sensors possess a huge potential and are most suitable in the context of biomedical application. When modified with different nanomaterials, they can offer a variety of biomolecules to be identified with great specificity and sensitivity.

In electrochemical sensor, the transducer converts biological event into an electrical signal. Two most commonly used parameters in electrochemical sensing are amperometric and potentiometric. In potentiometric, the analytical information obtained through biorecognition process is converted into potential, while in amperometric, constant potential current associated with reduction or oxidation of an electroactive species is monitored [3]. Therefore, they are employed extensively in disease diagnostics for the detection of suitable marker proteins, antibodies, DNA sequences or cells.

The development of novel diagnostic tools draws more attention towards point-of-care applications. This provides us with a major challenge of developing a new material in electroanalytical techniques that can specifically sense in vivo analytes. With the advent of nanotechnology, nanomaterial-based biosensors have shown immense possibility of diagnosis and detection of disease biomarkers more efficiently. Important advances in this aspect have been made with the utilization of different types of nanomaterials such as metal nanoparticles [4], magnetic nanomaterials [5], carbon materials [6], etc. to improve electrochemical signal of biocatalytic events occurring on the electrode surface.

Nanomaterials are characterized by excellent properties, like high surface area-to-volume ratio, good electrocatalytic nature (e.g. carbon-based nanomaterials) and enhanced adsorption capacity (e.g. gold nanoparticles). This leads to the fabrication of electrochemical sensors that exhibit improved sensitivity and selectivity [7]. Nanostructures, like nanowires (NWs), nanotubes (NTs), nanoparticles (NPs) and quantum dots (QDs) have been explored extensively for biosensing, since their size is comparable to the chemical and biological species to be sensed. Nanomaterials are employed in modifying electrochemical transducers so as to improve the transfer of electron in an analytical application and also provide biocompatible microenvironment to biomolecules. Recently, efforts are being made to use nanostructured modified electrodes for monitoring specific biological species in vivo [8] which opens up the possibility to detect a specific molecule in living organisms. So that real-time monitoring of some analytes like glucose can be implemented [9]. There is a need for the development of in vivo sensors to directly examine the nature of biological process, as in vitro sensing generally fails to completely explain the complexity of the living system. Different devices have been formulated which can implement real-time monitoring of biological events like muscular dystrophy, inflammatory events, infections or release of proteins in an in vivo environment as well. In vivo sensing requires sensitive instrumentation which can monitor signals inside living system. Detectors should be non-toxic and biocompatible and do not perturb the system.

Realizing the potential role of electrochemical sensor in diverse areas of biology and medicine, we have selectively reviewed here the recent advances in biomedical prospects of electrochemical sensor. Various modifications of electrodes have been made so as to increase the compatibility of biological species with the surface. Employing nanomaterials like carbon-based nanomaterials, metallic nanoparticles (such as silver and gold), metallic oxides, etc., in electrochemical biosensor has been discussed. We have also highlighted future perspectives and challenges related to this rapidly growing technology.

## Electrochemical Biosensor

To enhance the effectiveness of disease treatment, early diagnosis of disease is an important issue which needs to be resolved. Highly sensitive sensors are urgently required to measure extremely low level of markers and detect early stages of the disease, which will increase the survival rate of patients [10]. Existing diagnostic tests (e.g. glucose strips, ELISA) are not sensitive enough, and their detection limit corresponds to advanced stages of the disease. Faster, cheaper and miniaturised implantable devices are now desired. It will implement real-time monitoring of the diseased condition and make results available at patient bedside within few minutes [11, 12]. In this regard, electrochemical sensors are considered to be highly sensitive. They can easily be miniaturised and have fast analytical time compared with other conventional immunoassay techniques. And complex instrumentation system is also not required [13].

**Fig. 3:**
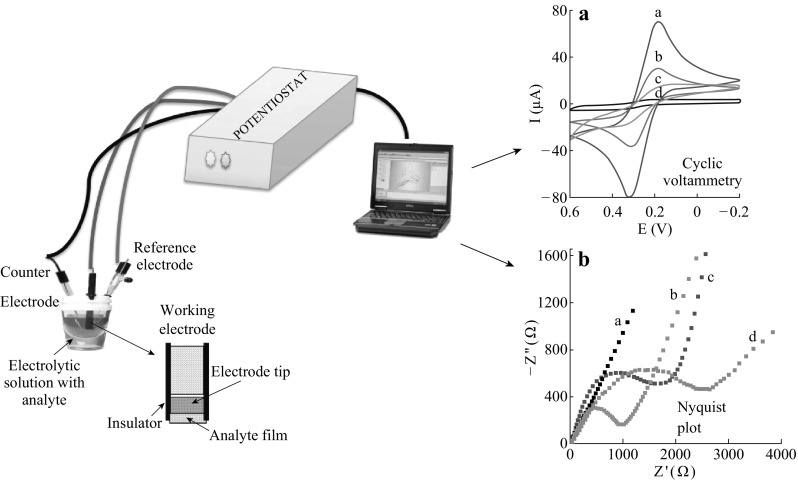
Illustration of electrochemical biosensor

**Fig. 4:**
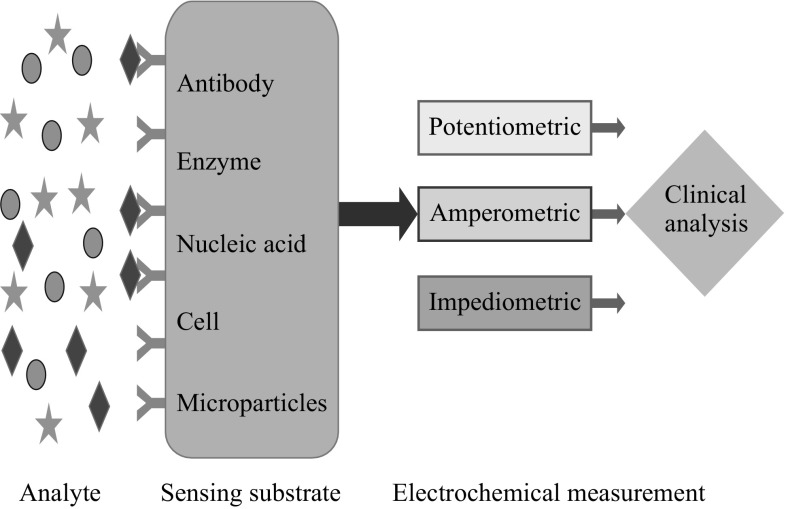
Electrochemical sensing of biological species

Electrochemical sensor is a tool that reads the chemical information of a sample and converts the data into an analytical signal. That information may be originated from the physical property of the system or from the reaction of a species present in that system. Data provided by the receptor unit are transferred to a transducer unit which converts them into an analytical form. Conventionally, a three-electrode system is employed in electrochemical biosensing for the target analytes as shown in Fig. 3. The working electrode is considered to play a key role in the redox process of an electrochemical cell. Different types of high-cost metal electrodes like platinum, mercury, gold and silver to low-cost glassy carbon, carbon paste and screen-printed electrodes are now being used as the working electrodes. Bioreceptor molecules like enzymes, nucleic acid, antibodies, dyes and metal ions are immobilised on electrodes for enhancing the signal and better recognition of analytes and biomarkers shown in Fig. 4. A potential is applied to the working electrode with respect to the reference electrode (Ag/AgCl, saturated calomel), while the counter electrode (platinum wire) is accustomed to complete the electrical circuit. On applying a negative potential, electron passes from the working electrode into the solution and reduces the analyte, whereas the reverse is obtained on applying a positive potential. The analysis of the reaction can be made through different modes like cyclic voltammetry (CV), differential pulse voltammetry (DPV) and square wave voltammetry (SWV). Techniques are applied for broad-spectrum behaviour analysis of target substance in electrolytic solution. Impedance spectroscopy (IPS) is also being utilized for target analyte detection where the increase in radius of semi-circle in Nyquist plot reflects the presence of target molecules. Different sorts of nanomaterials like graphene, nanotubes, silica-based NPs, metallic NPs, TiO_2_ and ZnO have shown excellent sensing performance in both sensitivity and selectivity with an extremely lower limit of detection (LOD) [14–16].

Electrochemical sensing has widely been explored to identify markers of different diseases like cardiac diseases, cancer, acquired immunodeficiency syndrome, hepatitis and urinary infections (Table 1) [17–22]. A variety of electrochemical analysis methods such as amperometric, voltammetric, conductometric and impedimetric are reported based on the disease biomarker. Miniaturised implantable electrochemical biosensors are now considered as an important tool for in vivo sensing of various metabolites like blood glucose, triglycerides and cholesterol to various protein biomarkers, bacteria and viruses without requiring patient intervention and its physiological state (rest, sleep, exercise etc) [23–26].

**Table 1:** Electrochemical diagnosis of different diseases based on their respective biomarkers

S. no.	Disease	Bioreceptors	Biomarker	Electrode modification	Detection limit	References
1.	Cancer	Antibodies	Carcinoembryonic antigen (CEA)	Glutathione-modified AuNPs	0.01 ng mL^−1^	[70]
			Prostrate-specific antigen (PSA)	Graphite modified by Au	0.5 pg mL^−1^	[22]
		Nucleotides	TP53 gene	Au	50 fM	[71]
		Cells	MCF7 cancer cells	Aptamer modifed	1000 cells	[72]
2.	Cardiac	Antibodies	Troponin T	Graphite powder	0.2 ng mL^−1^	[17]
			Troponin I	Graphene	4.5 pg mL^−1^	[50]
			Myoglobin	Didodecyldimethylammonium bromide-stabilized AuNPs	10 ng mL^−1^	[51]
			C-reactive protein	Macroporous Au	0.1–20 ng mL^−1^	[14]
		Cells	Platelet-derived microparticles (PMPs)	Graphene oxide	100 microparticles µL^−1^	[15]
3.	Hepatitis	Antibodies	Hepatitis B surface antigen	AuNPs	2.3 ng mL^−1^	[21]
			Hepatitis C	Graphite	1 ng mL^−1^	[73]
4.	AIDS	Antibodies	p24 antigen	AuNPs	0.01 ng mL^−1^	[19]
		Enzyme	HIV protease	Au	10 pg mL^−1^	[74]
5.	Urinary tract infection	Enzyme	Lactoferrin	UTI sensor array	104 cfu mL^−1^	[18]
6.	Malaria	Antibodies	Malarial antigen PfHRP2	ISE (27504-30; Cole-Palmer)	20 ng mL^−1^	[64]
7.	Nucleotide antibodies	Gliadin	Covalent attachment to Au coated with DT2	46 ng mL^−1^	[75]	
		tTG	Screen-printed carbon electrodes modified with MWCNT and AuNPs	2.45 U mL^−1^ for tTG IgA	[16]	

## Bioreceptors in Electrochemical Sensing

### Enzymes

Enzymes are considered to be an important biomarker for the analysis of different diseases through electrochemical detection. Enzymes are generally protein molecules of oxidase type that can selectively react with the target analyte. They can be easily immobilised on the electrode surface by physical adsorption, covalent bonding and various other techniques [27, 28]. Blood glucose measurement is generally done by three major enzymes: glucose-1-dehydrogenase (GDH), glucose oxidase (GO_x_) and hexokinase. Glucose oxidase (GO_x_)-modified electrodes are playing an important role in easy-to-use blood sugar testing [29]. Kang et al. and Shan et al. detected glucose by direct electrochemistry of GO_x_ on graphene, exploiting its excellent electron transfer property [30, 31]. The p24 HIV capsid protein was electrochemically sensed by CV using horseradish peroxidase (HRP)-labelled antibody-conjugated AuNP-modified glassy carbon electrodes (GCEs) and hydroquinone as a redox mediator [32]. Alpha-enolase is another metabolic enzyme that acts as a plasminogen receptor which works by the activation of plasmin. It is in the degradation of extracellular matrix. The enzyme level is upregulated in tumour cells supporting invasion of cancer. Enolase was detected using two antibodies: anti-enolase monoclonal antibody adsorbed on the electrode surface and polyclonal antibody labelled with AuNPs. The detection of this analyte was done through SWV with the LOD of 11.9 fg mL^−1^ [33]. The important feature of sensing through enzyme is its availability in highly pure form, their specificity for the substrates and ability to detect large number of analytes. The sensors fabricated by enzymes can be used continuously as they are unaltered at the end of the reaction. However, the drawbacks are their limited stability and activity dependence on various aspects such as temperature, pH, ionic strength and chemical inhibition (Table 1).

### Nucleic Acids

Nucleotide (DNA or RNA) sequences are also employed as biomarkers, and single-stranded DNAs are immobilised as biorecognition elements. If the complementary sequence is present in the sample, binding occurs and electrochemical response is generated. The detection occurs by complementary binding of nucleotides like adenine (A) to thymine (T) and cytosine (C) to guanine (G). DNA-based recognition elements, aptamers, whose function is analogous to antibodies, bind to the target and generate signal of recognition. Cancer marker tumour promoter (TP53) gene which encodes p53, a tumour suppressor protein, mutation in this gene leads to a variety of human cancers [34]. Discrimination between the mutated and normal gene was made using methylene blue-labelled short-hairpin molecular beacon as a recognition layer. The current was monitored through cyclic voltammetry by oxidation/reduction of methylene blue [35]. Nanomaterials like nanogold and quantum dots are also being used for conjugating DNA molecules, which makes this detection highly sensitive and more selective [36]. Electrochemical detection of human hepatitis B and papilloma viruses was carried out through impedance spectroscopy using AuNP-conjugated single-walled carbon nanotube (SWCNTs) [37]. Electrochemical DNA sensor based on graphene was developed by Zhou et al. They reported the simultaneous detection of different bases, and the electrodes were also capable of separating all four bases in both single-stranded DNA (ss-DNA) and double-stranded DNA (ds-DNA) [38]. A disadvantage associated with this system is their specificity and stability in electrolytic solution.

### Cells

Cells are also regarded as an important bioreceptor as they are highly sensitive to environment. They get easily immobilised on the surface of electrode and functions well as a biorecognition layer to frequently detect parameters like toxicity, stress and effect of drugs. In cell biorecognition, cell membrane recognises the element present in the solution such as aptamers, antibodies or small cell vesicles. Reports have been established on the role of electrochemical sensor in the detection of different cells like cancer, A549 cell line and bacteria cells [39–41]. Human umbilical vein endothelial cells (HUVECs) were immobilised on the electrode surface to construct an endothelial cellular biosensing system to detect nitric oxide through DPV [42]. Pond snail neuron cell membrane potential was monitored using glass microelectrodes to access the concentration of serotonin [43]. Cell-based potentiometric sensor for the detection of toxins was fabricated by attaching endothelial cells to a K^+^ selective membrane. When it is exposed to a specific class of compounds, the permeability membrane increases and more K^+^ ion can penetrate producing a potential response [44]. Detection of formaldehyde and cholanic acids has been done using immobilised yeast where any change in metabolism was detected via O_2_ electrode measurements or extracellular acidification rates [45, 46]. The disadvantage associated with the cell-based detection is its large size which may create steric hindrance in the system and the presence of undesirable enzymes. This obstructs its specificity and makes the results less reliable and ambiguous.

### Antibodies

Antibodies are protein molecules which are obtained from B-lymphocytes in any kind of antigenic stimulation. They are immobilised on the electrode surface through covalent bonds such as thiol, amide, ester, etc. Electrochemical immunosensor for prostate-specific antigen (PSA) detection has been developed using silver hybridized mesoporous silica nanoparticles (Ag@MSNs) as an electrode material and hydroquinone (HQ) as a mediator. Graphene sheet–methylene blue (GS-MB) nanocomposite was employed for fabricating an immunosensor to analyse PSA with a detection limit of 13 pg mL^−1^ [47]. Lu et al. reported detection of human chorionic gonadotrophin (hCG) by forming a sandwich-type immunosensor with AuNPs dotted CNTs–graphene composite having a detection limit of 0.034 ng mL^−1^ [48]. Specific monoclonal antibody against human cardiac troponin I (cTnI) was modified on AuNP-coated ITO electrode surface by self-assembly for the clinical detection of cTnI [49]. Another carbon nanofiber nano-electrode array is also explored for the detection of cTnI as low as 0.2 ng mL^−1^ [50]. Myocardial infarction biomarker like myoglobin was quantified through electrochemical nanosensors using AuNP/didodecyl dimethyl ammonium bromide (DDAB/Au)-modified electrode utilizing SWV [51]. Our group has recently reported graphene oxide-based electrochemical biosensor for detecting platelet-derived microparticles (PMPs), which are regarded as a major risk factor for thrombotic pathologies like acute myocardial infarction (AMI) and stroke. Graphene oxide was immobilised on electrodes along with PAC1 antibodies. Results indicated a progressive rise in the impedance of Nyquist plots with increasing concentration of PMPs in blood plasma sample. Blood obtained from patients diagnosed with acute myocardial infarction exhibited significantly higher values of circulating PMPs, thus validating the specificity and selectivity of the sensor [15]. Few limitations are associated with antibody-based electrochemical sensors, like the binding affinity and irreversible antigen–antibody interaction.

## In Vivo Applications of Electrochemical Sensors

In vivo electrochemical sensing is a well-established technique which offers real-time monitoring of analyte through implanted microelectrodes [52]. In general, these sensors were decorated by making amperometric changes that depict biological events like enzymatic activity.

### Glucose Sensors

In glucose monitoring, glucose oxidase is immobilised on the electrode surface to detect the electron transfer process. Glucose electrochemical sensors are embedded within the blood vessels which are directly linked to signal processing unit and implanted wires for supplying power. In this device, electrode is modified with glucose oxidase-conjugated biocompatible material and then covered with a selectively permeable membrane to reduce signal interference. Concentration of glucose is quantified by measuring oxygen (O_2_) consumption or hydrogen peroxide (H_2_O_2_) production via electrochemical oxidation or reduction occurring on the surface of the working electrode. Due to the advent of nanomaterials (metallic nanomaterials, carbon nanotubes, graphene, quantum dots, etc.), electrochemical sensors have gained large importance as nanomaterials largely retain the activity of enzymes or antibodies bound to them. In addition, they also facilitate the fast electron transfer between enzyme and electrode. Nanogold used in a glucose biosensor has shown sevenfold increases in the rate of electron transfer rate and a decrease in interference from O_2_. A needle-implantable in vivo glucose sensor with high sensitivity was prepared by using a nanoporous working electrode decorated with platinum nanoparticles [53]. The two implantable electrochemical glucose sensors (CGMS System Gold and GuardianTM system, from Medtronic MiniMed) have FDA approval.

#### Continuous Glucose Monitoring System (CGMS)

CGMS is a needle-type amperometric enzyme electrode which is implanted subcutaneously and coupled to a portable logger, from which data can be downloaded after up to 3 days sensing. The sensor is based on the conventional technology in which glucose oxidase is immobilised at a positively charged base electrode, detecting hydrogen peroxide production:

$${\text{Glucose }} + {\text{O}}_{2} \mathop{\longrightarrow}\limits_{{\text{Glucose\,oxidase}}}{}{\text{H}}_{2} {\text{O}}_{2} + {\text{gluconic\,acid}}.$$

For the measurement of glucose concentrations, the sensor is implanted in the subcutaneous tissue, through which interstitial fluid flows and the level of glucose is monitored [54, 55]. It is similar to normal measurement, but in conditions when glucose level changes rapidly, for example after a meal, these implantable devices are beneficial. The magnitude of changes has been recorded in needle-type enzyme electrodes in animal and human studies [56, 57]. An unpredictable drift and impaired response are two major problems associated with subcutaneously implanted electrodes.

#### GuardianTM System

It is designed so as to take readings from the patient for 3 days and must be calibrated with a self-monitoring blood glucose (SMBG) system for at least every 12 h [58, 59]. Lower and higher levels of glucose alerts are generally set, and the software is provided through which data can be downloaded and analysed.

#### Glucowatch Biographer

It extracts glucose through intact skin via reverse iontophoresis process. Hydrogel discs are used as electrolytes and reservoirs in which glucose is collected. GO_x_ is dissolved into hydrogel discs. When the reaction occurs, the peroxide concentration changes can be measured coulometrically. The total concentration of peroxide is related to the concentration of blood glucose. This system generally provides six readings h^−1^ and can be operated for 13 h before requiring replacement [60]. The major issues associated with the implanted glucose biosensors are instability, the foreign body response, protease activity, etc.

### Neurochemical Sensing

Analysing the brain’s extracellular chemical environment has the potential to provide a significant insight into neurotransmission, pharmacology and behaviour. Recently, the more significant applications of in vivo electrochemical sensing are in the field of neuroscience. As neurotransmitter signalling behaviour cannot be monitored effectively in in vitro assays, the development of in vivo electrochemical biosensors for the understanding of brain is important [61]. In vivo monitoring provides information about the working of the neural networks whether they are active or not. Because neurochemicals are electrically active, the electrochemical signals can be easily generated. Fast-scan cyclic voltammetry (FSCV) is an important electrochemical technique. It allows measurement of the release and uptake dynamics of endogenous monoamine level. This technique is used mainly to detect three major neurotransmitters, serotonin (5-HT), dopamine (DA) and norepinephrine (NE), as they can be oxidized at low voltages. By the incorporation of wireless data transmission with implantable sensors, real-time measurements of dopamine and serotonin level have been made freely in moving animals [62]. In another study, the kinetics of nitric oxide signalling in brain have been measured through in vivo electrochemical biosensors [63]. The in vivo measurement of rapid changes in the extracellular concentrations of l-glutamic acid in mammalian brain during normal neuronal activity or following excessive release due to episodes of anoxia or ischaemia in brain tissue has been made [64].

The most popular materials for in vivo electrochemical sensing are carbon, platinum, gold and iridium. These materials are often shaped into microwires with a microdisc-shaped tip, which can be mechanically polished. These probes have advantages of batch fabrication, high reproducibility of micro-scale features and flexibility to customize electrode recording site placement and substrate shape.

## Heavy Metal Ion Detection

Recent reports showed the detection of heavy metal ion in different types of diseases like cancer and malaria [65, 66]. Urine and blood is recognised as the best non-invasive method for monitoring a broad range of toxic metals ions, whose detection is important for detecting various diseases. This detection has been an issue due to protein competition and electrode fouling. Lead in urine has been detected using supermagnetic iron oxide nanoparticles (Fe_3_O_4_) functionalized with dimercaptosuccinic acid (DMSA) by stripping voltammetry [67]. Cadmium levels in urine primarily reflect the total body burden. Blood cadmium levels are indicative of recent exposure rather than whole-body burdens. The most sensitive targets of cadmium toxicity are the kidney, bone and lung cancer. Recently, Kudr and his group reported the presence of Zn, Cd, Pb and Cu ions in artificial blood plasma samples and Cd ion in chicken embryo by electrodeposition of mercury film over carbon tips [68]. Kensova and co-workers analysed the Cd concentration in blood using mercury electrodes with a detection limit of 0.002 µM [69]. Elevated lead concentrations in human blood are associated with damage to kidney, liver and gastrointestinal tract as well as the central nervous system [70]. The electrochemical sensors used in the detection of different heavy metal ions in various types of diseases is shown in Table 2.

**Table 2:** Electrochemical sensors in the detection of different heavy metal ions in various types of diseases

Diseases	Sample matrix	Analyte	Detection limit	Electrodes used	References
Cancer, lung infections, kidney disorder	Blood	Lead	0.001 µM	Hg	[68]
			1.2 µg dL^−1*^	Hg/screen-printed	[69]
			0.46 ppb	Hg microelectrode	[76]
			0.03 μg mL^−1^	Mercury film/carbon	[67]
			–	Bismuth	[77]
			0.23 ppb	Hg-plated pre-anodized screen-printed carbon	[78]
		Cadmium	0.002 µM	Mercury	[68]
			–	Bismuth	[78]
			0.06 μg mL^−1^	Mercury film/carbon	[67]
			0.1 nM	Nafion-coated mercury-plated glassy carbon	[79]
		Zinc	0.01 µM	Mercury	[69]
			0.6 μg mL^−1^	Mercury film/carbon	[68]
		Cooper	0.01 µM	Mercury	[69]
			0.02 μg mL^−1^	Mercury film/carbon	[68]
	Urine	Lead	0.5 ppb	GCE/ferromagnetic rod	[65]
			0.44 ppb	Hg microelectrode	[80]
Hyponatremia	Urine	Sodium	–	Ion-selective (ISE-27504-30; Cole-palmer)	[65]

* Lower limit of linear range in calibration

## Challenges and Future Aspects of Electrochemical Biosensors

Medical diagnostics require a rapid, accurate and portable system which could easily be available in patients’ bedside with real-time monitoring system. Currently, electrochemical sensing is facing some challenges which should be sorted out to get a highly sensitive and selective system for the diagnosis of diseases. Stability of the sensor is an important parameter for single-use electrodes and those which are to be used repeatedly. With the advances in point-of-care devices for diagnostic purposes, portability of the electrochemical analyser is also an important issue which needs to be resolved. Another important challenge for future development of electrochemical sensors is in vivo analysis of samples conveniently. In general, the ideal in vivo biosensor should be biocompatible, stable for longer period, sensitive and non-toxic to the host. A variety of approaches and techniques have been utilized to address the challenges of in vivo sensing. As many nanoparticles are biocompatible, the toxicity detected by other sensors can be minimised. Nanoparticles show less reactivity to proteins and do not have the capability of eliciting immune response. In addition, advances in the miniaturisation of the device, wireless power and data transmission all promise to reduce the invasiveness of many in vivo electrochemical sensors. High specificity of modern electrochemical assays could be achieved using biorecognition element of different small molecules (e.g. folic acid to detect cancer cells or more recently used aptamers). Sensor arrays need to be designed for detecting multi-analytes (metabolic markers such as glucose, lactate and uric acid). Non-invasive microfluidic biosensors, capable of extending the sizes of arrays and reducing the sample volume, should be developed to facilitate early detection and treatment of disease. The potential of electrochemical sensors is extremely promising for incorporating all these recent changes and thus driving force towards the development of point-of-care testing and monitoring of the disease. A wide range of excellent high-quality sensors have been developed in diverse areas like food industry, environmental pollution detection, heavy metal detection in water, etc. Significant advances have been made in the design and application of electrochemical sensors. Still changes need to be strengthened to focus on designing simple and cost effective sensors with improved sensitivity, response time and selectivity.

## Conclusions

Highly sensitive sensors are now required to measure extremely low level of biomarkers and detect early stages of diseases. In this regard, electrochemical sensors are considered to be the best candidate having fast analytical time, label-free nature and higher sensitivity and specificity. In this review, we addressed the recent advances in electrochemical sensors for their applications in biomedical field. Different modifications of electrodes in the context of enhancing compatibility of biological species against electrode surface have been discussed. Nanomaterials like carbon-based nanomaterials, metallic nanoparticles and metallic oxides are widely used as biomarkers in electrochemical biosensing. Besides, we have highlighted the use of miniaturised, non-toxic in vivo sensing devices which are widely used for analysing glucose and different neurotransmitters as clinical biomarkers. The future perspectives and challenges related to this rapidly growing technology were also discussed. Such major developments suggest that future interdisciplinary efforts will yield new generations of biosensors having a wide range of applications.
